# IUC22940-82 A 7-year review of robot-assisted vs laparoscopic radical nephrectomy at a UK district general hospital (2018-2024)

**DOI:** 10.1093/oncolo/oyaf248.045

**Published:** 2025-08-29

**Authors:** A Hossain, S Khan, A Albnhawy

**Affiliations:** Surrey and Sussex Healthcare, NHS Trust, United Kingdom; Surrey and Sussex Healthcare, NHS Trust, United Kingdom; Redhill, United Kingdom

## Abstract

**Background:**

Radical nephrectomy remains the gold standard for managing renal cell carcinoma (RCC) unsuitable for nephron-sparing approaches. While laparoscopic techniques are well-established, robotic surgery is increasingly adopted in district general hospitals. This study evaluates perioperative outcomes, complication profiles, and surgical trends in robot-assisted versus laparoscopic radical nephrectomy over 7 years.

**Methods:**

We conducted a retrospective review of 113 patients who underwent radical nephrectomy between 2018 and 2024 at a UK district general hospital. Fifty patients underwent robot-assisted, and 63 underwent laparoscopic procedures. Variables analyzed included patient demographics, estimated glomerular filtration rate (eGFR), operative time, TNM staging, complication rates (using the Clavien-Dindo classification), margin status, POSSUM risk scores, and time from diagnosis to surgery.

**Results:**

Among 113 patients, the mean age was 66.4 years in the robotic group and 64.8 years in the laparoscopic group. Preoperative eGFR was slightly lower in the robotic cohort (69.04 vs 73.86 mL/min/1.73m^2^). Mean operative time was longer for robotic surgeries (238 vs 191 minutes), though blood loss and transfusion rates were similar. Intraoperative complications were recorded in 2 robotic and 4 laparoscopic cases. Postoperative complications occurred in 13 robotic and 14 laparoscopic patients, with most classified as Clavien-Dindo Grade I or II. Margin positivity was slightly lower in robotic cases (2 vs 3), and robotic techniques were more commonly used for higher-stage tumors. The average time from diagnosis to surgery was 69 days, with some breaches of the NHS 62-day cancer target. POSSUM scores suggested higher surgical risk in robotic cases, possibly due to greater tumor complexity. Since its adoption in 2020, robotic nephrectomy has steadily increased, comprising 70% of all radical nephrectomies by 2024.

**Conclusion:**

Robot-assisted radical nephrectomy offers safe and comparable outcomes to laparoscopic surgery, with potential advantages in handling complex cases. Its increasing adoption reflects enhanced surgical ergonomics, improved precision, and institutional investment. Ongoing evaluation of surgical outcomes, timely access, and careful patient selection are key to optimizing the integration of robotic surgery in district general settings.

